# A Consideration of Some of the Relations of Climate to Tubercular Disease

**Published:** 1852-12

**Authors:** 


### ECLECTIC DEPARTMENT,

### AND SPIRIT OF THE MEDICAL PERIODICAL PRESS.

---

### *A Consideration of some of the Relations of Climate to Tubercular Disease.* By W. J. Burnet, M. D., Boston.

There are two prominent facts which have made the subject of the climatic relations of tubercular disease, one under active discussion among the medical men of this country and Europe during the last few years.

These are: first, the almost alarming increase of disease of this nature; and, second, the facilities of travel, so that climate can be easily and cheaply changed. The time has been when only a few thought about distant travel for health. But now, almost every one who at all values his life, can easily put himself in a more genial atmosphere and beneath an almost cloudless sky. With the attention thus directed, the questions are—*what* climate is to be sought; and what are the reasonable expectations as to its effect upon tubercular disease?

Of late there has been published quite a number of works upon the climate of those European and insular countries hitherto quite celebrated as resorts for invalids of this character; and, as the most dissimilar views have been advocated, there has arisen much confusion among medical men as to the correct answers of the questions above referred to. Some, in fact, have become thorough skeptics as to the benefit of any change of climate out of the latitude in which the invalid has been accustomed to live.

From among these works recently published may be mentioned two, viz., that of Dr. Pollock, appearing in the London Medical Gazette of last year; and that of Dr. Burgess, not long since separately published. Both are upon the climate of Italy, and are well calculated to lessen the enthusiasm of invalids for a land which has always been made more sunny by the pens of poets than the favor of nature. I have no doubt that the conclusions of these men, and especially those of Dr. Pollock, upon the climate of southern Europe, are correct in the main; and as they were addressed to the English people, will no doubt lead many English physicians to hesitate before advising their usual migration.

But in this country, a misapplication and sometimes a misinterpretation of these and similar opinions, has led very many physicians to be quite skeptical as to the real benefit to be derived by northern invalids, from a change of residence into the southern and more sunny states. This skepticism seems to be yearly increasing—and there can be but little doubt that it is as mischievous as it is really unfounded. It is certainly quite desirable that clear and distinct opinions should be entertained by northern physicians upon a subject fast getting to be one of such paramount importance. I make this remark, because I think that the reason of their doubts of climatic influence, is plain; in other words, that the cause of their unfortunate experience is becoming well understood. It is, that the climate has not been thoroughly tried. To make a clear and full statement of the whole matter, I will say that I am convinced that the shifting migratory course, South in winter and spring, and North the rest of the year, usually advised and followed, is an erroneous and mischievous one; and that if a northern consumptive can reasonably expect any benefit from this change of climate, this benefit will be obtained only from a continued southern residence for several years.

There is a grave error in thinking that, if one goes South in late autumn, and remains there until late spring, and then returns North to pass the summer and early autumn, he keeps himself in the train of favorable climate influences. It is not so; and the error is concealed in the fact that a summer at the North does not make a southern climate. This leads me to some considerations upon the peculiarities and differences of the northern and southern climates of this country.

As to the New England climate, it seems quite clear, that, taken as a whole, there is something in it highly predisposing to the development of tubercular disease. Not only do we see this disease here constantly peering out from hereditary predispositions, but the cases are quite numerous in which it seems purely indigenous, being engrafted upon an untainted stock. It is true that this may be said of other countries having an intemperate climate, but very far from the extent of what I think is true of New England. Statistics can be produced to show, that, take the whole year through, pulmonary diseases—inflammation of the mucous membrane of the air-passages—constitute a very large proportion of the disease. In fact, the tendency of disease here seems to be quite toward the pulmonary organs. Aside from the evidence of general observation, this statement has a very significant support in the fact, that in cases presenting some obscure aspects, the suspicion of the intelligent physician is quickly fastened upon the lungs, and an examination of the chest is made; thus showing that where outstanding local or temporary causes are absent, one is almost unconsciously led to suspect insidious disease referable to ever-constant general agencies.

An unequal fluctuating climate, in any latitude, tends to produce these effects. But the climate of New England, besides having this inequality and diversity in a very marked degree, possesses other characteristics having a great influence. Its atmosphere is dry and stimulating, and during the greater part of the year of a low temperature considering the latitude. The effect of such an atmosphere upon a sound constitution is highly bracing, leading to a mental and corporeal activity quite inconsistent with endurance and longevity. It is probably not an incorrect opinion that many of the moral and physical peculiarities of New England people, included under the terms enterprise and action, may be traced to these agencies.

In such an atmosphere, the constant vicissitudes of the temperature render the functions of the skin imperfect, thus increasing the liability of congestions of the mucous membrane; and this mucous membrane, from the fact that it is ever in contact with an irritating medium, is generally that of the air-passages. On this account, mainly, the urgency of these conditions is considerably lessened by the use of flannel next to the skin; the importance of which worn in summer as well as winter, is now well recognized.

On the whole, New England climate has little in it that is sedative at any long season of the year. The winters are broken and unsteady, especially so on the sea-board, and it is only in the northern inland portions that there is that constant cold which has a far more favorable influence. The character of New England spring weather is too well known to need comment. Nothing could be more uncertain and less reliable. The months of May and June frequently change places, and one is not sure of warm weather until into July. As for the summer months, it is a great mistake, as I have before said, to suppose that they furnish a climate like that of the South. There is, to be sure, heat enough, but it is unsteady, and during July and August the thermometer not unfrequently falls 30° or 40° in a few hours. Intensely hot as it is frequently in midday, yet at midnight, if one is exposed, it is rare that over-clothes are not the more comfortable.

But a fact more significant than all the rest as to the influence of our summer weather, is that our consumptives do not generally improve in it; on the other hand, they lose ground. This is generally attributed to the depressing influence of the heat. No doubt there is much in this, for the heat is here often very intense; but more is probably due to the sudden and wide changes of temperature. That this is the correct version of the matter, would seem to be indicated by the influence of our early autumn weather, which is far the best and most genial we have. There is generally a season, commencing about the first of September, and continuing until the early frosts of October, when the weather of new England may be said to be truly fine. The atmosphere is warm and dry, presenting a hazy, quiet aspect, and the light wind is generally from the W. or S. W. It is then that we have those dreamy days that come and go so quietly as scarcely to leave a ripple-mark —reminding one of the sunny skies of the pine-lands of Georgia and South Carolina. Every one, and especially those out of cities, has felt the soothing, sedative influence of this weather.

It is well known that during this weather, our consumptive and other pulmonary invalids improve. The functions of their skin are more active, and the urgency of the cough and all the other pulmonary symptoms is decreased. The expectoration is less purulent, the appetite improved, and the spirits, strength and flesh increased. In many instances the improvement is as unexpected as it is remarkable—and there is often a melancholy pleasure in thus observing this temporary improvement, brightened as it always is by the patient with a thousand delusive hopes.

This short season is the only weather in New England with which I am acquainted, that is really favorable to consumptive invalids.* And in its

* The fine weather of a New England June has always been insisted on and highly recommended. But of late years this does not appear to have been true — for it has been unsettled, and often colder and more uncomfortable than May. If one can trust the testimony of elderly people, it would seem that in this and other respects, the climate has changed very perceptibly in the last quarter of a century. Now, they affirm, the winters have not that steady severe cold as formerly, but are more open and broken, running into the spring; and this last, in its turn, usurping a portion of summer.

favorable influence, and at the same time in its resemblance to that of the pine-lands of the South, there may be drawn something more than a hint as to the real agency of southern climate upon diseases of this nature. But broad as this hint is, it is not usually taken; or if so, not in time. For many invalids in the second stage of consumption, improved as they have, do not perceive the wisdom in taking means to continue in this same climate, bnt delude themselves with the hope that they will be well enough to remain North during winter; or, if they conclude to go South, defer it until they are obliged to, having two or three "colds upon their lungs."

The peculiarities of a southern climate as bearing upon its benefit to consumptive invalids, are far from being referable alone to its elevated temperature. I refer here to the alluvial and pine-land portion of Georgia and South Carolina. It has other characteristics, which, though less well understood, are not the less important as to effects. The atmosphere has a decidedly sedative, soothing influence, which, due to whatever causes it may be, has a very desirable effect upon the mucous membrane of the air-passages—and this effect, once commenced, is not likely to be disturbed by sudden vicissitudes of temperature. There the general tendencies of disease seem to be changed; and that, too, *from* the thoracic *to* the cutaneous and abdominal organs; and it is through these changed relations that the cure is to be effected. But a fact more worthy of notice than all the rest, is the almost complete exemption from phthisis of the native inhabitants of this section of the country. It is true that consumption is there found; but a careful inquiry has shown that in almost every instance it had been immigrated either directly or indirectly. Other diseases, such as those of a miasmatic character, those of the intestinal canal and its appendages, seem to exist in the place of those of a tubercular nature; and were we better acquainted with that curious yet important subject—*the antagonism of diseases*—we might, perhaps, better understand how these relations are effected.

That these relations of disease are based upon climatic influences, might be here shown in many ways; but I will mention one fact, observed by myself, which is quite indicative. In northern and upland Georgia, the soil and aspect of the country quite resembles that of New England. There, as in New England, the primitive geologic rocks appear; and it has for a long time been remarked, that nowhere South is the climate so much like that of New England as in this section. The diseases follow in the same train, for they are pre-eminently those of the pulmonary organs. Consumption, lung fever, bronchitis, are common, and this, too, at the apparent exclusion of the diseases of the low and pine-land regions.

An additional fact of the same bearing, and which may here be mentioned, is, that, even in the pine-land country of upper South Carolina, a very severe winter (as the last, for instance) is quite productive of pneumonia or lung fever with those inhabitants living on creeks or in damp spots. The construction of their houses is little calculated to shield them from the adversities of cold and damp; and thus situated, it is rather a moticeable fact, that the disease assumes an acute form, exactly as is true of the Irish of New England, in whom tubercular tendencies are not common; whereas, among our native inhabitants, acute pneumonia is rather a rare disease, the pulmonary affections being generally of a more chronic and insidious nature.

If such are the influences of climate upon comparatively healthy constitutions, we should naturally infer that its tendency would be toward arresting the development of tubercular disease, and favoring that condition of the general system leading to a permanent cure.

That this is so, I fully believe, and think it can be tolerably well shown, imperfect as the state of inquiry has hitherto been.

But if we sought proof in the results of migratory invalids, our case would truly be a poor one. If climate is to work a change, it is foolish to expect that that change will be effected unless the individual gets acclimated. It is, therefore, to the results of those cases of tubercular disease where the residence has been permanent, that we are to look for a correct version of the matter.

In my intercourse with many intelligent plysicians at the South, many cases were described to me, in which individuals from the North, having phthisis in its first stage, had taken up their permanent residence there. Their pulmonary symptoms gradually disappeared, and now they are quite free from them, enjoying a very fair share of health. In the same manner, also, several cases were described to me, in which the disease had far advanced in the second stage—a cavity of small cavities having been produced in one of the lungs. These individuals remained there permanently, settling down into a quiet life. They recovered so as to enjoy tolerable health—the cure taking place, as indicated by physical signs, much in the way Laennec has described, by the partial cicatrization of the cavities, which yielded a blowing, dry, amphoric sound. In one of these instances the young man felt so much restored after a few years, that he hazarded a return to New England for a permanent residence. But in less than a year he was seized with a violent and unexpected hæmorrhage, and died soon after of ordinary phthisis.*

It is to be regretted that statistics upon this subject have not been made out; but as the matter now stands, the conviction left in the mind of the medical inquirer and observer is full and clear.

There is another fact, vouched for by an intelligent physician of Georgia, and which should be mentioned in this place. He affirmed to me that the negroes of Maryland and Northern Virginia, affected and broken down by pulmonary trouble, and perhaps scrofula, as shown in enlarged glands, &c., if sold to the Georgia and other far Southern planters, soon improved, losing their symptoms, quite often recovering, and growing strong and fat.

I was also struck with the fact of the long duration of phthisis with those negroes of the South, who, from quite ill conditions of life, had contracted the disease. It seemed to run a light, lengthy form, although perhaps fatal in the end. I recall to my mind one instance, where I examined the chest of a negro having tuberculosis of the apices of both lungs, and a cavity in the left one. To the physician with me I declared that he would die in three months. But he affirmed that he would live two to three years, and that, as property, this probability of life would be admitted.

But I need discuss this matter no farther. It now remains for me, in conclusion, to make a few general remarks.

The view I advocate is, that if a consumptive can reasonably expect benefit

* In citing these facts, I trust I shall not be misunderstood. I am very far from advocating the doctrine that all who have consumption in the first and second stages, can get well by living permanently at the South; but I do advocate that if benefit in these cases can be reasonably hoped for by this change of climate, this change should be permanent.

from a southern climate, his residence there must be permanent and not migratory.

Besides the arguments already adduced in support of this view, it may be worth while to notice the testimony given me by those physicians residing in the winter resorts of northern consumptives. Generally, they say, they (the invalids) do not arrive there until actually driven by the cold weather of the North. As soon as the warm, delightful weather of April has come, and they are, if at all, in a fair way for permanent improvement, they are uneasy about their return North; and the occurrence of two or three quite warm days in succession, soon settles their determination. By early May they have left, looking much better than when they came. The ensuing winter they appear again, but it is evident they have lost ground during their absence; they return home again in early spring as before, and here often is the end of their migrations. Others, having the disease in a more chronic form, appear regularly for many years; but at last are not seen or heard of again.

I am aware that invalids, on going South, expect too much in the way of climate. They picture in their minds cloudless skies over a land of the cypress and myrtle, and which will immediately effect their restoration. I need scarcely say that in this they are doomed to disappointment; and so will it always be, until the opinion is fully recognized—that it is not sunny skies that will alone benefit them, but rather a continuation under the aggregate of the influences of the climate.

At the present day numerous objections are raised by northern physicians against this southern migration. One class disapprove of it on the ground, both of the incurability of the disease, and a disbelief in warm climate, based upon an ill-digested theory, partly chemical and partly medical. Another class, and much the more numerous, although avowing a belief in southern climate, nevertheless quite object to the migration on the ground of humanity. They cry out against what they call the cruelty of sending people away from the comforts and attentions of home—and that too with a wide possibility to die among strangers. In its place they advise the patient to remain among the comforts of home—occupying a large chamber, which by various arrangements is to have a southern or summer atmosphere.

There is some force in a part of this objection, for sometimes there is great inconsiderateness in urging patients away. But, taken as a whole, it is not valid. Certainly no judicious person would advise the going away of a patient unable to bear the journey, or whose end is not far distant. But the conveniences of modern travel have taken away the former terrors of the transit. The journey now is easy and of short duration, and with mail and telegraph one can feel quite near home. With these conveniences there seems little necessity for the immuration of an invalid in a chamber—obliged all the while to take sedative medicines for cough—and however many and complete the home-comforts, yet in a fair way to depress the nervous system, and enervate the whole body.

In no disease is there so much danger of over-medication as in consumption. Experience has shown, that as a disease primitively of the nutrition, our object must be to strengthen the nutritive function, and to spare every unnecessary dose of medicine into the stomach, the tone and power of which, must be carefully nursed by proper food. I need scarcely say that these relations cannot be carried out by a winter's residence at the North, however favorable the circumstances.

In cases where the symptoms are not immediately threatening, and the patient has remaining considerable physical power, so as to be about in an easy way without fatigue, it will generally, I think, be judicious to advise, at least a winter's residence at the South, where one can be under the influence of pleasant days, and drink in balmy air instead of cough mixtures.

As to a summer's residence at the South, beside the objection of its being unnecessary, there is another generally urged—the enervating effect of its excessive heat. This objection is not well founded, and rests more upon ideas of a more southern latitude than any thing else. As to degree of heat, the mercury certainly rises higher in the New England than in the Southern States. For in these last it rarely exceeds 90°, even in the hottest season. It is true that the hot season is long, and, in the low sandy regions, its effect is quite depressing. But possessing such a variety of climates as does South Carolina and Georgia, the invalid need not thus be endangered, for there are resorts midway between the low and the mountainous parts of both of these States, where the summer climate is indescribably fine, having, perhaps, no equal in this or any other country.*

But in advocating the necessity of a permanent southern residence for the consumptive, I should be willing to do so only with some exceptions. There is a class of patients, generally of the so-called lymphatic and bilious temperaments, who bear heat badly; and what they gain in a decrease of local symptoms, they lose in general strength. I need scarcely say that this class of cases everywhere is the most intractable, and least amenable to treatment. It belongs to the judicious physician to perceive the relations of such cases, and advise accordingly.† As to variety of climate and climatic advantages, the United States are certainly more highly favored than any country. If this fact is known generally, it is not appreciated. No invalid need cross the water; for in our own borders, among our own people, who speak the same language as ourselves, we can, by a journey of less than eighty hours, be in a clime certainly not surpassed by any of the old world. Dissatisfied as the English are fast getting with their "sunny Italy," or their "beloved Madeira," it may not be regarded improbable that, when the communication shall have become easier and more direct, they will exchange these for the sunnier spots of Carolina and Georgia.

Boston, September 13, 1852.—*Boston Med. and Surg. Journal.*

---

*Tourniquets on Railroads.*—Several of the English railroad companies, and especially the Midland, have a supply of these very important instruments on board, which are often eminently serviceable in case of accident, till a surgeon can be called. They should be kept by the conductors of our American roads also. Many a death occurs from hæmorrhage in cases of

---

* Such is the character of climate of Greenville and its neighborhood in South Carolina, and of Stone-Mountain, in Georgia. In fact, there can be little doubt that the climate of both of these States is far better in summer for invalids than in winter.

† In this connection I may make a remark having an unrestricted application. It is, that in a disease so precarious as consumption, if an individual residing at the South is doing well, the wisdom of letting well alone and remaining there, should be recognized, however late in spring the time may be. They should not act up to the dictates of a common theory, before they have tested its value in their cases, by individual experience.

